# Silencing the CSF-1 Axis Using Nanoparticle Encapsulated siRNA Mitigates Viral and Autoimmune Myocarditis

**DOI:** 10.3389/fimmu.2018.02303

**Published:** 2018-10-08

**Authors:** Ingmar Sören Meyer, Carl Christoph Goetzke, Meike Kespohl, Martina Sauter, Arnd Heuser, Volker Eckstein, Hans-Peter Vornlocher, Daniel G. Anderson, Jan Haas, Benjamin Meder, Hugo Albert Katus, Karin Klingel, Antje Beling, Florian Leuschner

**Affiliations:** ^1^Internal Medicine III, University Hospital Heidelberg, Heidelberg, Germany; ^2^DZHK (German Centre for Cardiovascular Research), Partner Site Heidelberg-Mannheim, Heidelberg, Germany; ^3^Institute of Biochemistry, Charité - Universitätsmedizin Berlin, Corporate Member of Freie Universität Berlin, Humboldt-Universität zu Berlin, and Berlin Institute of Health, Berlin, Germany; ^4^DZHK (German Centre for Cardiovascular Research), Partner Site Berlin, Berlin, Germany; ^5^Cardiopathology, Institute for Pathology and Neuropathology, University Hospital Tuebingen, Tuebingen, Germany; ^6^Max-Delbrueck-Center for Molecular Medicine Berlin, Berlin, Germany; ^7^Internal Medicine V, University Hospital Heidelberg, Heidelberg, Germany; ^8^Axolabs GmbH, Kulmbach, Germany; ^9^David H. Koch Institute for Integrative Cancer Research, Massachusetts Institute of Technology, Cambridge, MA, United States; ^10^Cardiovascular Division, Department of Medicine, Brigham and Women's Hospital, Boston, MA, United States; ^11^Department of Chemical Engineering, Massachusetts Institute of Technology (MIT), Cambridge, MA, United States

**Keywords:** inflammation and immunmodulation, innate immunity, cytokines, monocytes/macrophages, RNA interference, virus, infection-immunology, myocarditis

## Abstract

Myocarditis is an inflammatory disease of the heart muscle most commonly caused by viral infection and often maintained by autoimmunity. Virus-induced tissue damage triggers chemokine production and, subsequently, immune cell infiltration with pro-inflammatory and pro-fibrotic cytokine production follows. In patients, the overall inflammatory burden determines the disease outcome. Following the aim to define specific molecules that drive both immunopathology and/or autoimmunity in inflammatory heart disease, here we report on increased expression of colony stimulating factor 1 (CSF-1) in patients with myocarditis. CSF-1 controls monocytes originating from hematopoietic stem cells and subsequent progenitor stages. Both, monocytes and macrophages are centrally involved in mediating tissue damage and fibrotic scarring in the heart. CSF-1 influences monocytes via engagement of CSF-1 receptor, and it is also produced by cells of the mononuclear phagocyte system themselves. Based on this, we sought to modulate the virus-triggered inflammatory response in an experimental model of Coxsackievirus B3-induced myocarditis by silencing the CSF-1 axis in myeloid cells using nanoparticle-encapsulated siRNA. siCSF-1 inverted virus-mediated immunopathology as reflected by lower troponin T levels, a reduction of accumulating myeloid cells in heart tissue and improved cardiac function. Importantly, pathogen control was maintained and the virus was efficiently cleared from heart tissue. Since viral heart disease triggers heart-directed autoimmunity, in a second approach we investigated the influence of CSF-1 upon manifestation of heart tissue inflammation during experimental autoimmune myocarditis (EAM). EAM was induced in Balb/c mice by immunization with a myocarditogenic myosin-heavy chain-derived peptide dissolved in complete Freund's adjuvant. siCSF-1 treatment initiated upon established disease inhibited monocyte infiltration into heart tissue and this suppressed cardiac injury as reflected by diminished cardiac fibrosis and improved cardiac function at later states. Mechanistically, we found that suppression of CSF-1 production arrested both differentiation and maturation of monocytes and their precursors in the bone marrow. In conclusion, during viral and autoimmune myocarditis silencing of the myeloid CSF-1 axis by nanoparticle-encapsulated siRNA is beneficial for preventing inflammatory tissue damage in the heart and preserving cardiac function without compromising innate immunity's critical defense mechanisms.

## Introduction

Myocarditis and its sequela, dilated cardiomyopathy, are leading causes of heart failure and sudden death in young adults (1). While various agents may provoke cardiac inflammation, viral infections are the most common trigger of myocardial inflammation in the Western world. Although various viruses are putative invaders of heart tissue, most of our knowledge on disease pathology comes from infection with enteroviruses, in particular CoxsackievirusB3 (CVB3). CVB3 had been reported among the most prevalent pathogens causing viral myocarditis in North America and Europe in the past (2, 3). Mouse models using different strains with divergent susceptibility for cardiotropic CVB3 elegantly reflect human disease with highly diverse disease outcome (4, 5). The hereditary susceptibility involves a certain immune-anchored genetic phenotype leading either to altered virus control and/or to induction of deleterious immunopathology (6–8). Severe virus-induced inflammation can result in a subsequent loss of self-tolerance against cardiac proteins, which contributes to additive auto-destructive activity of infiltrating cells and exaggerates heart tissue damage (9, 10). Cardiac myosin is such a crucial autoantigen in both human and murine virus-induced myocarditis (9). Administration of cardiac myosin or its pathogenic epitope in combination with an adjuvant induces experimental autoimmune myocarditis (EAM) in mice, a model that mimics certain aspects of myocarditis and heart failure in humans (11).

Treatment options for patients with myocarditis are sparse and both conventional immunosuppressive as well as anti-viral approaches have not yielded the desired results in clinical trials (12). Recent data suggest that it is not the presence and/or replicative activity of invading viruses in the myocardium that determines outcome, but the virus-triggered abundance of infiltrating leukocytes is an independent risk factor (13, 14). At the acute state of myocarditis in mice, the majority of accumulating leukocytes in inflamed heart tissue are CD11b^+^ monocytes and macrophages (15, 16). Consistently, the presence of CD68^+^ macrophages is a diagnostic hallmark for human myocarditis (3). Infiltration of immune cells is cytokine/chemokine-dependent. Consistent with previous findings (17), we have demonstrated that not CVB3-mediated cytotoxicity itself, but the overwhelming cytokine response initiated by viral PAMPs is responsible for disease severity. Lower pro-inflammatory cytokine/chemokine production during the early phase of infection paralleled in reduced inflammatory heart tissue damage and protected mice from cardiac failure (14). As monocytes and macrophages are key players that secrete pro-inflammatory and pro-fibrotic cytokines thereby exacerbating acute and chronic inflammatory injury during myocarditis (4, 18), effector molecules that modulate their differentiation, activity, and cytokine secretion might be putative drug targets for myocarditis. We have previously described the precise targeting of inflammatory monocytes and their precursors by optimized lipid nanoparticles which were encapsulated with siRNA directed against CCR2 or CD115 (19, 20). Injection of mice with these nanoparticles resulted in rapid blood clearance, accumulation in spleen and bone marrow, and localization to monocytes (19).

Here, we demonstrate RNA sequencing data obtained from endomyocardial biopsies of patients with myocarditis indicating a significantly increased production of Colony Stimulating Factor 1 (CSF-1). The development of monocytes depends on CSF-1 (21) and its receptor CSF-1R/CD115. CSF-1 can be expressed and produced by various cells including monocytes themselves (22). Local production of CSF-1 stimulates tissue-resident macrophage proliferation and reduces apoptosis, thereby influencing cellular survival (23). CSF-1R is expressed on monocytes, macrophages, dendritic cells and their precursors, including “granulocyte-macrophage progenitors” (GMP), “monocyte-macrophage DC progenitors” (MDP) and “common monocyte progenitors” (cMoP) (24, 25). CSF-1 receptor signaling is a well-described mechanism that leads to monocyte production from progenitors and stimulates mature monocytes screwing them into a pro-inflammatory state (26). Based on this, we hypothesized that disruption of the CSF-1 axis in myeloid cells attenuates heart muscle inflammation and the resulting organ damage during myocarditis. Using mouse models of CVB3-induced myocarditis and experimental autoimmune myocarditis, we have found that silencing of CSF-1 upon treatment of mice with CSF-1 siRNA encapsulated nanoparticles substantially mitigated inflammatory heart muscle damage leading to less fibrosis formation and improved heart muscle function without the risk of exacerbating direct viral pathology.

## Materials and methods

### Study approval

All subjects gave written informed consent in accordance with the Declaration of Helsinki. The study protocol was approved by the ethic committee of the Medical Faculty—University of Heidelberg—project 390/2011 “Central biobank of Department Internal Medicine III for research on molecular and genetic markers in patients with cardiovascular disease.”

### RNA-seq analysis, read processing and mapping

Patient enrollment and biomaterial processing for RNA-seq analysis of heart biopsies was performed as previously described (27). In detail, biopsy specimens were obtained from the apical part of the free LV wall during cardiac catheterization using a standardized protocol. Biopsies of 1- to 2-mm diameter were immediately washed in ice-cold saline (0.9% NaCl), transferred and stored in liquid nitrogen until RNA extraction. After diagnostic workup of the biopsies (histopathology), the remaining material was used to isolate RNA with an Allprep Kit (Qiagen). RNA purity and concentration were determined using the Bioanalyzer 2100 (Agilent Technologies) with a Eukaryote Total RNA Pico assay for RNA from biopsies. Sequencing libraries were generated using the TruSeq Stranded Total RNA Sample Preparation Kit with Ribo-Zero Human/Mouse/Rat from Illumina, adhering to the standard protocol of the kit. Sequencing was performed using 2 × 75 bp paired end sequencing on an Illumina HiSeq2000 instrument. For transcriptome analysis, raw read files were mapped with STAR v2.4.1c5 using GRCh37/hg19 and the Gencode 19 gene model (http://www.gencodegenes.org/). Read counts were generated with help of subread's feature counts program 6 (subread version 1.4.6.p1), using uniquely mapped reads only (28). Normalization was performed with help of rlog-normalization (29). RNA seq data were deposited to the public repository Gene Expression Omnibus (GEO) - NCBI, accession number GSE120567. RNA seq data for DCM patients are partially demonstrated in (30).

### Differential gene expression- and gene set enrichment analysis

Differential gene expression analysis of RNA-seq data was carried out within the RStudio framework using the edgeR package (31). Gene set enrichment analysis was performed with KEGG gene sets.

### Histology and immunohistochemistry

Human endomyocardial biopsy tissue and murine tissue was stained as described elsewhere (32). For AVM, paraffin embedded organ tissue sections were stained with hematoxylin/eosin (HE) or Masson's trichrome according to standard protocols. Immunohistochemical stains for CSF-1 (rabbit polyclonal, abcam), T lymphocytes (CD3 and CD4) and mononuclear phagocytes (Mac-3) was performed as previously described (32). For EAM, hearts were excised 30 days after primary immunization. Hearts were rinsed in PBS, fixed in 10% formaline for 24 h and embedded in paraffin. Serial 5 μm sections were stained with Masson's trichrome staining to quantify fibrotic tissue formation. Severity of EAM was evaluated according to a 6-tier scoring system as previously described (19, 20). All slides were counterstained with hematoxylin. Sections were mounted with Pertex mounting media (Medite). Slides were viewed with a Zeiss Axioskop 40 microscope.

### Candidate identification of CSF-1 siRNA

Lysates of several murine cell lines were tested on CSF1 expression. NIH-3T3 cells showed high CSF1 expression, are readily to be transfected, and were therefore used in candidate identification experiments. siRNA loaded lipid-based nanoparticles were generated by Axolabs GmbH (Kulmbach, Germany) as previously described (19). siRNA targeting CSF-1 receptor (CSF-1R, CD115) is described elsewhere (33). To generate siRNAs that target the CSF-1 transcript, NIH 3T3 cells were transfected with siRNAs targeting CSF-1 or non-targeting control siRNA complexed with Lipofectamine2000 Transfection Reagent at 5 and 50 nM final concentration in quadruplicates. Values for CSF-1 were normalized to GAPDH and related to the mean value of three different control siRNAs (100% expression). Optimal siRNA concentration yielding most efficient knockdown of CSF-1 production was obtained with RNA transfections starting at 100 nM in 6-fold dilution steps down to 10 fM. CSF-1 siRNAs that showed the best knockdown in both the dual concentration screen and the concentration response curve screen were used for nanoparticle encapsulation and *in vivo* experiments.

### Induction of acute viral myocarditis (AVM) and experimental autoimmune myocarditis (EAM)

For induction of AVM, 5–7 weeks old male A.BY/SnJ mice were infected intraperitoneally (i.p.) with 5 × 10^5^ PFU CVB3 (cardiotropic Nancy strain) provided by Klingel (15) and Rahnefeld et al. (32). Original breeding stocks for A.BY/SnJ mice were purchased from the Jackson Laboratory. For EAM, male BALB/c were purchased from Janvier (Saint-Berthevin, France). Myocarditis was induced by subcutaneous injection of an emulsion containing 150 μg myosin peptide SLKLMATLFSTYASAD (PSL GmbH, Heidelberg, Germany) supplemented with complete Freund's adjuvant (CFA) (Sigma-Aldrich, Taufkirchen, Germany) and 5 mg/ml *Mycobacterium tuberculosis* H37Ra (Sigma-Aldrich, Taufkirchen, Germany). Directly after the initial immunization, mice were injected with 500 ng pertussis toxin (Sigma-Aldrich, Taufkirchen, Germany) i.p. Seven days after the primary immunization, mice received a second subcutaneous injection of 150 μg myosin peptide supplemented with CFA and complemented with *Mycobacterium tuberculosis*. All mice were housed under standard laboratory conditions with a 12-h light-dark cycle and access to water and food *ad libitum*. For AVM, the protocol was approved by the Committee on the Ethics of Animal Experiments of Berlin State authorities [G0034/16]. EAM experimental protocols were approved by the institutional review board of the University of Heidelberg, Germany, and the responsible government authority of Baden-Württemberg, Germany (project number 35-9185.81/G-209/12). All mouse studies were carried out in accordance with the recommendations in the Guide for the Care and Use of Laboratory Animals of the German animal welfare act, which is based on the directive of the European parliament and of the council on the protection of animals used for scientific purposes. All efforts were made to minimize suffering.

### *In vivo* silencing of CSF-1 during AVM and EAM

The optimal CSF-1–targeted siRNA (siCSF-1) was scaled up for *in vivo* studies. For viral myocarditis, mice were intravenously treated with 0.5 mg/kg nanoparticle encapsulated siLUC or siCSF-1 immediately prior to CVB3 infection and 2, 4, and 6 days after infection. For EAM, nanoparticle treatment started 14 days after the primary immunization with myosin peptide. Animals received four i.v. injections of 0.5 mg/kg siCSF-1 or siLuciferase (LUC)-nanoparticles (control siCD115) per week.

### Evaluation of knockdown efficacy of siCSF-1

Male BALB/c mice received single injections of 1.5 mg/kg lipid-based nanoparticle containing either siLUC or siCSF-1 on three consecutive days. Animals were sacrificed 24 h after the third injection. Bone marrow cells were isolated and prepared for flow cytometry-based sorting of monocytes, which were identified as Lin^−^(CD90;B220;CD49b;NK1.1;Ly6G,Ter119);F4/80^−^; CD11c^−^; CD11b^+^. Cell sorting was performed on a FACS ARIAII (BD Bioscience, Heidelberg, Germany). RNA from sorted cells was isolated using Trizol (Life Technologies, Darmstadt, Germany). Knockdown efficacy was evaluated using quantitative real-time PCR. Gene expression was normalized to HPRT. The following primers were used: CSF-1: TCCCATATGTCTCCTTCCATAAA (fwd), GGTGGAACTGCCAGTATAGAAAG (rev); CD115: CGAGGGAGATCTCAGCTACA (fwd), GACTGGAGAAGCCACTGTCC (rev). HPRT: GTCAACGGGGGACATAAAAG (fwd), TGCATTGTTTTACCAGTGTCAA (rev). For the AVM model, spleen tissue was isolated 8 days after virus inoculation and tissue homogenization was performed using a lysis buffer containing 20 mM HEPES, 1 % (v/v) Triton X-100, 4 mM EDTA, 1 mM EGTA, 5 mM TCEP, 50 mM NaF, 5 mM NaPP, 2 mM Na-*o*-vanadate and Complete® protease inhibitor cocktail (Roche). Western blot analysis was performed following standard procedures. After blocking with 5% milk/PBS-Tween at 4 °C overnight, membranes were probed with the primary antibody α-CD115 (ab32633, Cell Signalling) and α-actin (Merck Millipore). The bound primary antibodies were detected using IRDye800CW labeled goat anti-mouse secondary antibodies in conjunction with an Odyssey CLx infrared imaging system (Li-Cor Biosciences, Bad Homburg, Germany).

### Echocardiography

Cardiac function and morphology of mice with AVM were assessed with a VisualSonics Vevo 770 High-Frequency Imaging System with the use of a high-resolution (RMV-707B; 15–45 MHz) transducer during anesthesia with 1.5–2% isoflurane. Temperature and ECG were continuously monitored. For the EAM model, echocardiography was performed in conscious animals on a VisualSonics Vevo 2100 30 days after the first immunization. Standard imaging planes, M-mode, and functional calculations were obtained. For AVM, the parasternal long-axis four-chamber view of the left ventricle (LV) was used to guide calculations of percentage fractional shortening, ventricular dimensions and volumes. M-mode echocardiographic images were recorded at the level of the papillary muscles from the parasternal short-axis view. An experienced reader blinded to treatment performed all measurements. Ejection fraction (EF) and fractional shortening (FS) were calculated based on M-mode measurements.

### Flow cytometry

Flow cytometric analysis was performed 8 days after infection in AVM and 21 days after the first immunization in EAM. Single cell suspension of bone marrow, spleen and heart tissue were prepared as previously described (34). Hearts were flushed with PBS and homogenized in RPMI 1,640 medium (Biochrom) containing 10% (v/v) fetal calf serum (FCS) (Biochrom), 1% (v/v) penicillin/streptomycin (Pan Biotech), 30 mM HEPES, 0.1 % (w/v) collagenase type 2 (Worthington) and 0.015% (w/v) DNase I (Sigma-Aldrich) at 37°C at 800 rpm for 30 min. Afterwards, 10 mM EDTA was added. Cells were washed with PBS and passed through a 70 μm cell strainer as described in reference (35). For the identification of myeloid cells, cell suspensions were stained with a cocktail of PE-conjugated anti-mouse antibodies targeting hematopoietic lineage markers (B220 for B cells (RA3-6B2, BD Bioscience), CD90.2 for T cells (53-2.1, BD Bioscience), CD49b for NK cells (DX5, eBioscience), NK-T/NK Cell Antigen for NK cells (U5A2-13, BD Bioscience) and Ter-119 for erythroid cells (TER-119, BD Bioscience)) and fluorescent-dye conjugated antibodies against the following cell surface markers: CD45.2 (104, Brilliant Violet 711™, BioLegend), CD11b (M1/70, PE-CF594, BD Bioscience), Ly6G (1A8, PerCP/Cy5.5, BioLegend), Ly6C (HK1.4, Pacific Blue™, BioLegend), CD11c (N418, Brilliant Violet 510™, BioLegend), I-A[b] (AF6-120.1, FITC, BD Bioscience) and F4/80 (BM8, APC, BioLegend). Cells were stained in PBS containing 2% FCS, 2 mM EDTA for 20 min at 4°C. For the identification of lymphoid cells, cell suspensions were stained with fluorescent-dye conjugated anti-mouse antibodies against CD45.2 (104, Brilliant Violet 711™, BioLegend), CD3e (145-2C11, PerCP/Cy5.5, BioLegend), CD4 (RM4-5, V500, BD Bioscience), CD8a (53-6.7, Pacific Blue™, BD Bioscience), B220 (RA3-6B2, FITC, BioLegend) and CD19 (6D5, APC, BioLegend). The antibody staining was followed by a cell viability stain (Fixable Viability Dye eFluor® 780, eBioscience) according to the manufacturer's protocol.

Monocytes were identified as Lin^low^ (CD90;B220;CD49b;NK1.1;Ly6G,Ter119), F4/80^low^, CD11c^low^, CD11b^high^; or Fixable Viability Dye^low^, CD45.2^high^, CD11b^high^, (B220, CD90.2, CD49, NK-T/NK Cell Antigen, Ter-119)^low^, Ly6G^low^, SSC^low^, F4/80^low^ and CD11c^low^ and further differentiated according to Ly6C-expression. Inflammatory monocytes express high levels of Ly6C and patrolling monocytes express low levels of Ly6C. Macrophages were identified as Fixable Viability Dye^low^, CD45.2^high^, CD11b^high^, Lin^low^, Ly6G^low^, SSC^low^, F4/80^high^ and CD11c^low/high^. Dendritic cells were identified as Fixable Viability Dye^low^, CD45.2^high^, CD11b^high^, Lin^low^, CD11c^high^ and MHC II^high^ (compared to isotype control). Neutrophils were identified as Fixable Viability Dye^low^, CD45.2^high^, CD11b^high^, Lin^low^, Ly6G^high^ and SSC^high^. B cells were gated as Fixable Viability Dye^low^, CD45.2^high^, CD3^low^, B220^high^ and CD19^high^. T cells were gated as Fixable Viability Dye^low^, CD45.2^high^, B220^low^, CD3^high^ and either CD4^high^ or CD8^high^. For identifying proliferating GMPs mice received two s.c. injections of 1 mg/kg Bromdesoxyuridin (BrdU) 12 and 24 h before the animals were sacrificed. BrdU was stained using BrdU flow kit (BD Biosciences). Proliferating GMPs were identified as (CD90;B220;CD49b;NK1.1;Ly6G;CD11b;CD11c;IL-7R;Sca-1)^low^ and (CD117;CD34;CD16/32;BrdU)^high^.

For the assessment of quantitative data, 123 count eBeads (eBioscience) were used according to manufacturer's protocol. Data were acquired on a FACS Verse (BD Biosciences, Heidelberg, Germany) or on a LSR II (BD Bioscience) and analyzed with FlowJo v10.0 software (FLOWJO, Ashland, United States). Reported cell numbers were normalized to the weight of total hearts, yielding the number of respective cell fraction per mg tissue.

### Determination of viral load in heart tissue

Plaque assays were performed in triplicates on sub-confluent green monkey kidney cell monolayers as described recently (32). *In situ* hybridization of CVB3 RNA was performed using probes generated with the DIGoxigenin (DIG) RNA labeling kit (Roche) and the pCVB3-R1 plasmid. Plasmid cDNA was linearized with SmaI (36); all other steps were conducted as previously described (37). DIG-labeled CVB3 RNA was detected using a horseradish-peroxidase-conjugated DIG antibody (Roche 1:100). HistoGreen (Linaris) was used as a substrate. All slides were counterstained with hematoxylin.

### High-sensitive (hs)-troponint (TnT)

Blood was sampled by facial vein puncture and collected in a heparinized capillary. Thereby obtained plasma was diluted 1:15 in PBS. hs-TnT was determined by the electrochemiluminescence method (ECLIA; Elecsys 2010 analyzer) according to the method described in reference (38).

### Statistics

Statistical analysis of the data was performed in GraphPad Prism v6.00/v.700 for Windows (GraphPad Software, La Jolla, California, United States). Logarithmic data (virus titer, semi-quantitative RNA quantification) measured on a linear scale was transformed logarithmically prior to data plotting and data analysis. Data summary is indicated on plots as mean ± SD unless stated otherwise. Unpaired *t*-tests were used for two group comparisons. If samples had unequal variances (determined by an *F*-test), an unpaired *t*-test with Welch's correction was used. For multiple group comparison unequal variance versions of ANOVA (1-way or 2-way ANOVA) were performed followed by a Sidak-Holm's multiple comparison test. The significance threshold for all tests was set at the 0.05 level.

## Results

### Increased abundance of CSF-1 in heart muscle tissue during myocarditis/inflammatory cardiomyopathy

**Figure 1:**
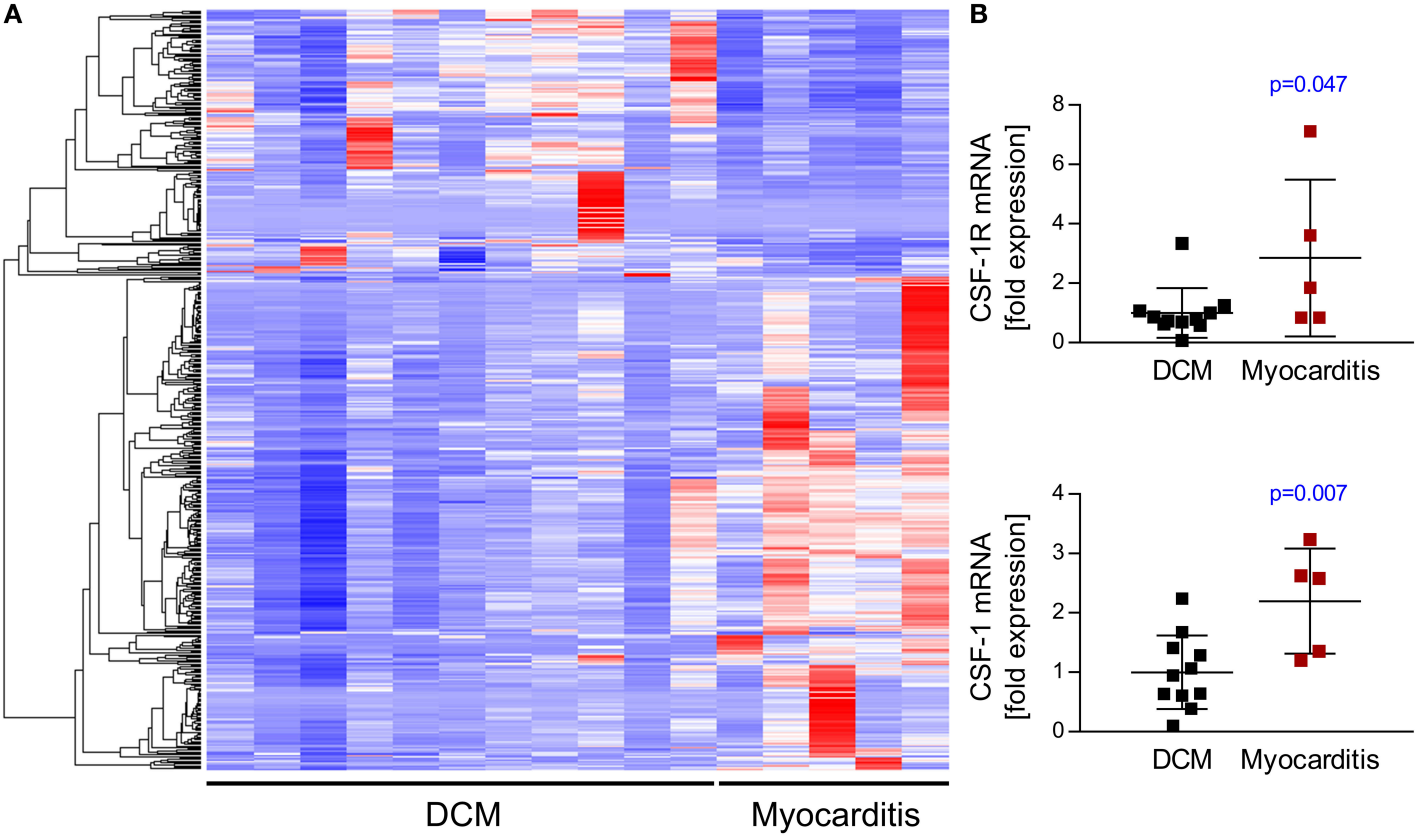
Transcriptome analysis of endomyocardial specimen from patients with dilated cardiomyopathy and myocarditis. RNA-Sequencing data from endomyocardial biopsies obtained from patients with clinically diagnosed myocarditis or dilated cardiomyopathy as a control were analyzed for relative expression of different gene sets. **(A)** RNA-seq analyses revealed differential expression of 1963 genes. Heatmap depicts top 500 differentially expressed genes hierarchically clustered by using Euclidean distance measures. **(B)** CSF-1 and CSF-1R expression taken from RNA-seq data. Unpaired *t-*tests were used; *p*-values are indicated on the graph and significant differences (*p* < 0.05) are marked with blue color.

**Figure 2:**
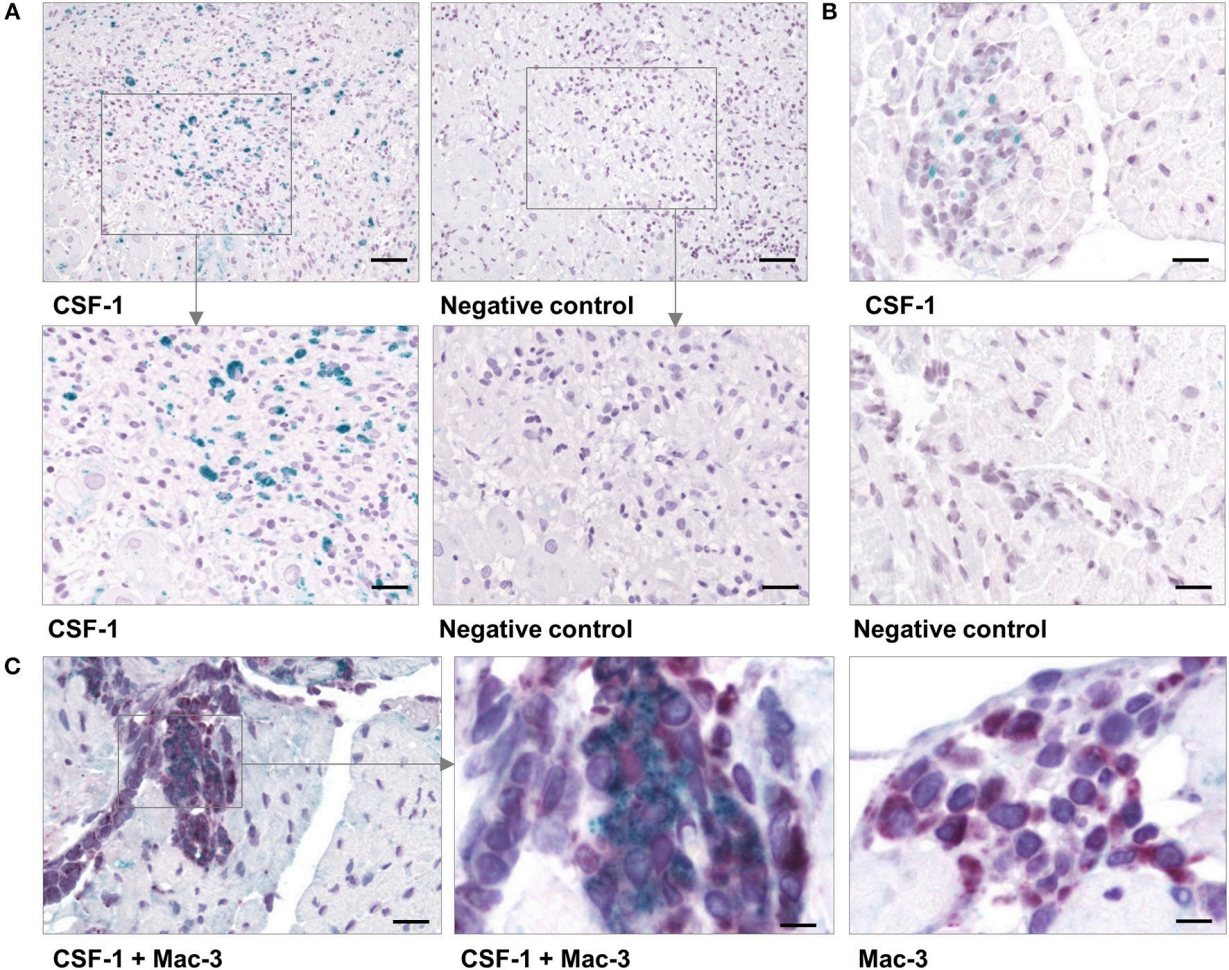
CSF-1 production in cardiac tissue during viral myocarditis. Paraffin-embedded tissue sections from endomyocardial biopsies that had been obtained from patients with acute myocarditis were stained by immunohistochemistry. **(A)** Representative micrographs stained with an anti-CSF-1 antibody [left column] or with a secondary antibody only [right column] are depicted. top: scale bar = 120 μm; bottom: scale bar = 60 μm. **(B)** Heart tissue sections were obtained from CVB3-infected A.BY/SnJ mice on day 8 *p*.i. Representative micrographs of anti-CSF-1 stained heart tissue are shown [scale bar = 36 μm]. **(C)** In addition, cardiac sections from mice were double-stained with an antibody directed against Mac-3 (red) [left: scale bar = 36 μm; center: scale bar = 12 μm] and against CSF-1 (green). As control, Mac-3 stained tissue sections were counterstained omitting the anti-CSF-1 directed antibody [right: scale bar = 12 μm].

RNA-seq analyses revealed that myocarditis results in a diverse transcriptional response in the patient's heart tissue. We observed 1963 differentially expressed genes in biopsies taken from patients with acute myocarditis vs. patients with a non-inflammatory dilated cardiomyopathy (DCM) (Figure 1A). Gene set enrichment analysis (GSEA) revealed that a decent amount of differentially expressed genes participates in inflammatory processes (especially cytokines and cytokine receptors) and differentiation of hematopoietic cell lineages (Table 1). Myocarditis leads to a massive infiltration with immune cells into heart tissue. Monocytes represent the most prominent leukocyte population both during virus-mediated and experimental autoimmune myocarditis (16, 32). Monocyte production and maturation is strongly dependent on CSF-1 and CSF-1R, and, both effector molecules were identified in two gene sets mentioned above. Our data indicate a pronounced up-regulation particularly of CSF-1 and CSF-1R in endomyocardial specimen from patients with myocarditis/inflammatory cardiomyopathy (Figure 1B). Immunohistochemical stain of heart tissue from patients with myocarditis revealed CSF-1 expressing cells only within inflammatory foci, with a strong focus on mononuclear immune cells (Figure 2A). Altogether, these data argued toward a significant contribution of monocytes/macrophages to the cardiac CSF-1 expression, which we found in patients with inflammatory heart disease.

**Table 1:** Top 10 C2 curated gene sets (KEGG Database) significantly enriched in human biopsies.

**Gene set name (KEGG database)**	**Description**	***p*-value**	**FDR *q*-value**
CYTOKINE_CYTOKINE_RECEPTOR_INTERACTION	Cytokine-cytokine receptor interaction	2.11E−20	3.93E−18
SYSTEMIC_LUPUS_ERYTHEMATOSUS	Systemic lupus erythematosus	5.61E−19	5.22E−17
CELL_ADHESION_MOLECULES_CAMS	Cell adhesion molecules (CAMs)	1.3E−17	8.07E−16
HEMATOPOIETIC_CELL_LINEAGE	Hematopoietic cell lineage	2.08E−15	9.67E−14
VIRAL_MYOCARDITIS	Viral myocarditis	5.19E−14	1.93E−12
NEUROACTIVE_LIGAND_RECEPTOR_INTERACTION	Neuroactive ligand-receptor interaction	2.81E−13	8.71E−12
LEISHMANIA_INFECTION	Leishmania infection	4.67E−13	1.24E−11
COMPLEMENT_AND_COAGULATION_CASCADES	Complement and coagulation cascades	2.62E−11	6.1E−10
RIBOSOME	Ribosome	1.91E−10	3.96E−9
CHEMOKINE_SIGNALING_PATHWAY	Chemokine signaling pathway	2.61E−10	4.64E−9

*RNA-Sequencing data of endomyocardial biopsies from patients with clinically diagnosed myocarditis or dilated cardiomyopathy as a control were analyzed for relative expression of different gene sets. Indicated are the names, description and p-values (with FDR-adjustment) of the most relevant gene sets*.

Local production of CSF-1 stimulates tissue-resident macrophage proliferation and reduces apoptosis (23). In addition to this function and a direct role of CSF-1 during monocyte development, it might also influence pro-fibrotic processes under inflammatory conditions. Since inflammation and fibrosis are hallmarks of inflammatory heart disease, we aimed to investigate the pathophysiological influence of CSF-1 with respect to manifestation of inflammatory heart tissue injury. First, we determined CSF-1 production in a mouse model of CVB3-induced myocarditis. We performed immunohistochemical stains to evaluate CSF-1 abundance during viral myocarditis. Consistent with our findings in patients, CSF-1 production was increased within inflammatory foci at the acute state of myocarditis in mice (Figure 2B). Since monocytes/macrophages represent the major infiltrating cell population in acute myocarditis, it is very likely that these cells are also involved in CSF-1 production. By double labeling immunohistochemistry we found CSF-1 protein expression in a part of Mac-3 positive mononuclear phagocytes within the cardiac inflammatory lesions (Figure 2C).

### Nanoparticle-encapsulated siRNA effectively downregulates CSF-1 production in monocytes

**Figure 3:**
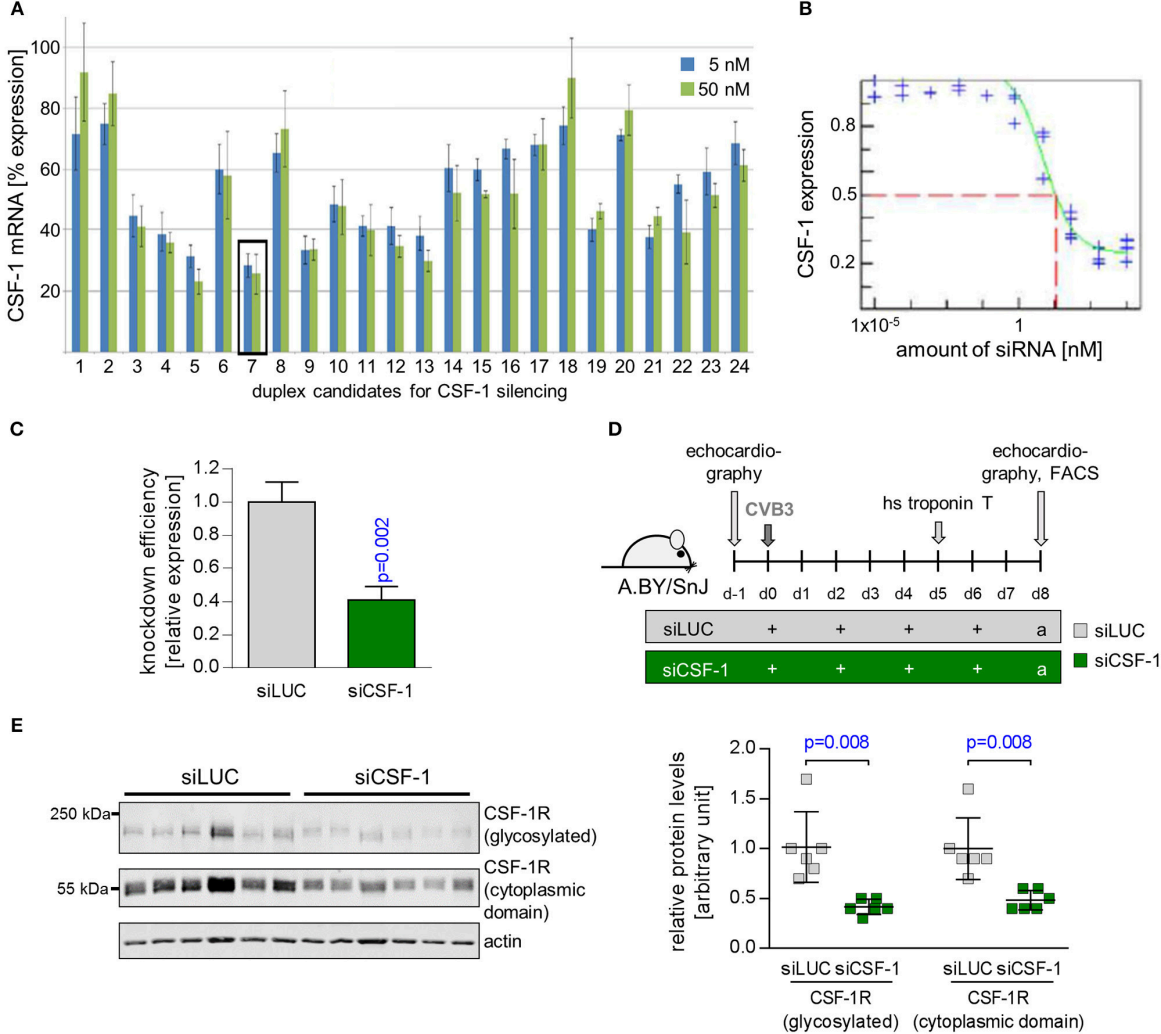
Suppression of CSF-1 production by siRNA-encapsulated nanoparticles. **(A)** NIH-3T3 cells were transfected with 24 siRNA candidates directed against CSF-1. siRNA #7 was most efficient to reduce CSF-1 mRNA expression both at 5 and 50 nM and was selected for further studies. **(B)** CSF-1-directed siRNA # 7 was titrated and the respective CSF-1 knockdown efficacy was determined. **(C)** Knockdown efficacy of nanoparticle-encapsulated CSF-1 candidate siRNA pool 7 after injection into naive Balb/c mice (*n* = 3). **(D)** A.BY/SnJ mice were intravenously treated with nanoparticle encapsulated siRNA targeting either luciferase (*n* = 7 siLUC, gray color) or CSF-1 siRNA #7 (*n* = 8 siCSF-1, green color) directly prior to CVB3 inoculation. siRNA treatment was repeated after 2, 4, and 6 days. **(E)** The overall efficacy of siCSF-1 treatment during AVM as indicated by the presence of CSF-1-R)-positive cells was monitored by Western blot analysis of spleen tissue homogenates (*n* = 6 mice per group) 8 day after virus inoculation. CSF-1R (high molecular weight band) and the cytoplasmic domain of CSF-1R (around 55 kDa) are depicted. Fluorescence was quantified by the Image Studio Lite Ver 5.2 software. Signal intensity was normalized to actin and is depicted as relative expression levels compared to siLUC-treated mice in the bar graph. Unpaired *t-*tests were used; *p*-values are indicated on the graph and significant differences (*p* < 0.05) are marked with blue color.

CSF-1 can be expressed and produced by various cells including monocytes themselves (22). siRNA encapsulated in lipid-based nanoparticles has been shown to effectively downregulate target genes in monocytes and their lineage progenitors (19, 20). Furthermore, *in vivo* knockdown of CSF-1R and monocyte depletion with nanoparticle-encapsulated siRNA has recently been demonstrated for ischemic heart disease (33). Thus, in order to investigate the pathophysiological function of CSF-1 production by monocytes/macrophages on inflammatory tissue damage during myocarditis, we decided to use a nanoparticle-encapsulated siRNA approach to target CSF-1 production in myeloid cells. To identify siRNAs leading to highly efficacious suppression of CSF-1 production, 24 different siRNAs targeting CSF-1 were investigated regarding their influence on CSF-1 mRNA levels *in vitro* (Figure 3A). Six different siRNAs, which yielded optimal *in vitro* suppression of CSF-1 mRNA production, were further investigated for their knockdown efficacy. Next, we screened the respective CSF-1 directed siRNA regarding to the concentration-dependent knockdown efficacy (Figure 3B) and selected the most efficacious siRNA for *in vivo* nanoparticle studies. Naive BALB/c mice were intravenously inoculated with 1 mg/kg of this nanoparticle-encapsulated siRNA (termed siCSF-1) on three consecutive days. Expression of CSF-1 was found to be effectively downregulated in monocytes that were sorted from spleen of siCSF-1 treated mice and further evaluated by quantitative PCR analysis (Figure 3C). Since pathogens are frequently involved in the pathogenesis of myocarditis (5), as a next step we set up an experimental approach to decipher the CSF-1 axis using nanoparticle-encapsulated siRNA in a mouse model of virus-mediated myocarditis. A.BY/SnJ mice with high hereditary susceptibility for the development of acute viral myocarditis (AVM) were treated with siCSF-1 or respective controls directly prior to infection with cardiotropic CVB3 (Nancy). siCSF-1 treatment was repeated every other day until mice were sacrificed 8 days after infection at the respective peak of infiltration in heart muscle (Figure 3D) (6–8). Following this protocol, we monitored the abundance of CSF-1 receptor levels in spleens of infected mice, which allowed us to conclude on the efficiency of siCSF-1 treatment during infection. Consistent with virus-mediated mobilization of monocytes/macrophages from bone-marrow sources, viral infection resulted in increased CSF-1R levels in the spleen. In siCSF-1-treated mice, we found reduced CSF-1R levels being indicative of suppressed myeloid cell mobilization upon siCSF-1 injection during AVM (Figure 3E).

### siRNA-mediated knockdown of CSF-1 attenuates virus-mediated pathology

**Figure 4:**
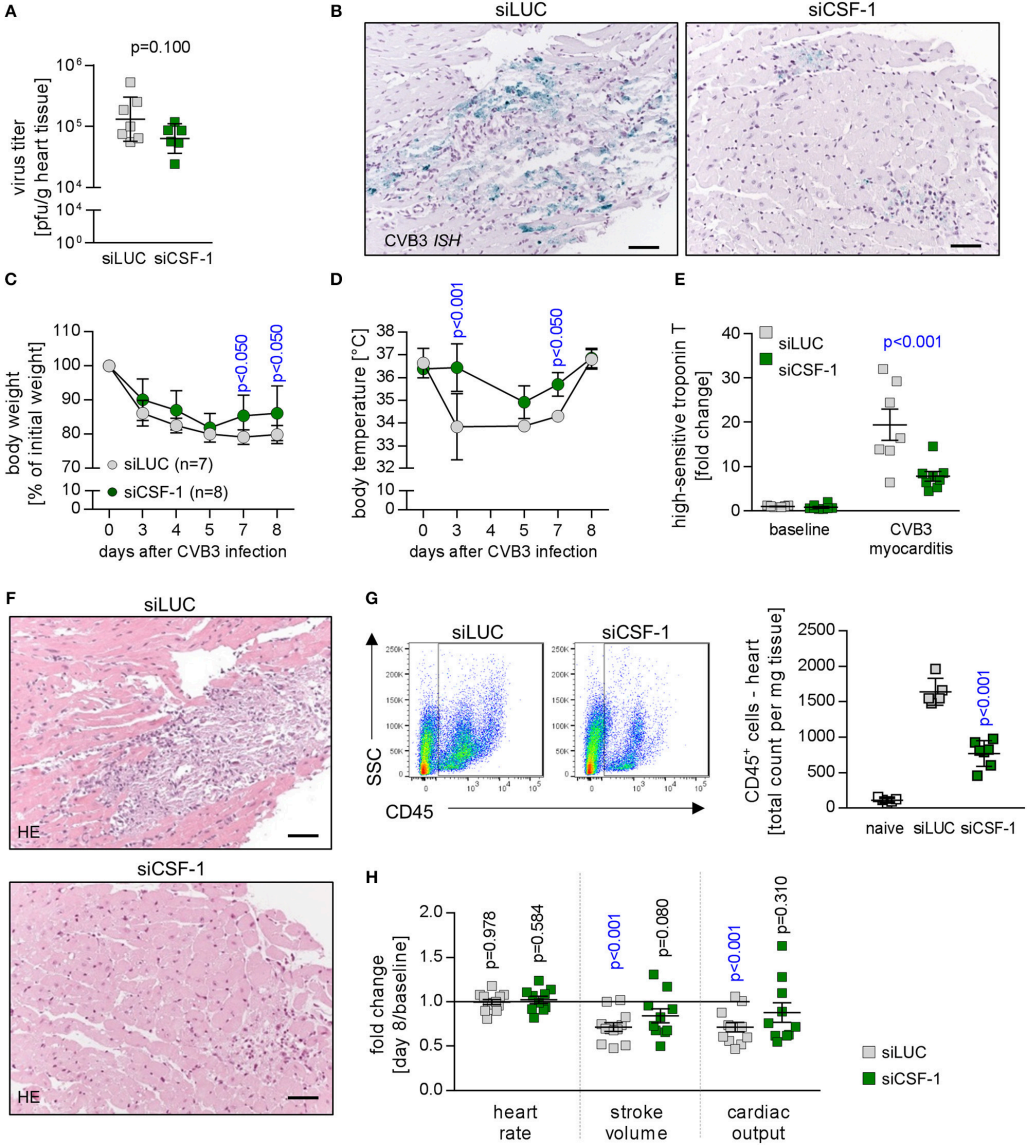
Depletion of CSF-1 attenuates virus-mediated pathology. Mice with AVM were subjected to CSF-1 siRNA treatment as indicated in Figure 3D. **(A)** Infectious virus particles were determined in heart tissue homogenates by plaque assay. Data summary is mean ± SEM. A student‘s *t*-test was conducted and the *p*-value is shown. **(B)** To localize viral RNA in infected heart tissue, *in situ* hybridization for the detection of the CVB3 genome was performed and slides were counterstained with hematoxylin/eosin. Representative micrographs are depicted (scale bar = 60 μm). During viral infection, mice were monitored for body weight **(C)** and body temperature **(D)** at the indicated points in time. Dots represent mean ± SEM. Repeated measurements versions of two-way ANOVA were performed followed by a Sidak- Holm multiple comparison procedure. P values are indicated (blue color indicates *p* < 0.05; only significant results are depicted on the graph). **(E)** To assess injury of cardiomyocytes prior to peak of inflammation, blood was sampled 5 days after infection by facial vein puncture and high sensitive (hs) troponin T plasma levels were determined. Obtained results were normalized to the results obtained with blood samples from non-infected siLUC-treated mice and are depicted as fold changes. Data summary is mean + SD. Repeated measurements two-way ANOVA was performed followed by a Sidak-Holm's multiple comparison test and the p value is depicted. **(F)** After sacrificing mice 8 days after infection, heart tissue sections were stained with hematoxylin/eosin. **(G)** To quantify cell infiltration, single cell suspensions of heart tissue obtained from naive mice (uninfected mice that did not receive siRNA treatment; white bars, *n* = 4) as well as AVM and siRNA-treated mice (siLUC: gray squares, *n* = 7; siCSF-1: green squares, *n* = 8) were stained with CD45 antibodies to quantify total leukocyte count in the heart. **(H)** Cardiac function was assessed by echocardiography prior to CVB3 infection in A.BY/SnJ mice (baseline) by an experienced and blinded investigator. Mice were allocated to respective groups: siLUC and siCSF-1. In all CVB3-infected mice, echocardiography was repeated 8 days after CVB3 infection (siLUC *n* = 16; siCSF-1 *n* = 17 mice). Data were analyzed regarding putative alteration during AVM in the respective treatment groups (day 8 after infection vs. baseline measurements of the same cohort). Relative changes of stroke volume, heart rate and cardiac output compared to baseline measurements were calculated for each group and these fold changes are depicted for siLUC and siCSF-1-treated groups. One-sample *t*-tests were performed to compare baseline measurements and values obtained 8 days after infection. All p values are depicted, *p* < 0.05 are in blue color.

Since we found reduced mobilization of monocytes/macrophages in siCSF-1-treated mice during CVB3 infection, this mouse model allowed us to delineate the pathophysiological role of CSF-1 production particularly by monocytes/macrophages during viral myocarditis. First, we questioned whether siCSF-1 treatment manipulated the viral load during AVM. The viral burden as reflected by the amount of infectious viral particles (Figure 4A) was not substantially influenced by siCSF-1 in heart tissue at the acute state of infection. Thus, targeting the CSF-1 axis represents a safe approach regarding to control of virus dissemination and replication in A.BY/SnJ mice. During virus-mediated myocarditis, there is a strong spatial-temporal relation between virus-induced cellular injury and the emergence of inflammatory foci in heart tissue (39). Likewise, viral genome abundance as detected by CVB3 *in situ* hybridization was spatially connected with high-grade inflammation and most impressive in siLUC-treated mice (Figure 4B). Since CVB3 does not only target the heart, but also replicates in the pancreas, we also determined the magnitude of virus-induced pancreas destruction and found similar tissue injury in both siLUC- and siCSF-1-treated groups (Figure S1).

Next, CVB3-infected mice that received siCSF-1 or siLUC as a control were followed for global signs of acute infection. siCSF-1 treatment profoundly attenuated overall virus-induced pathology as represented by significantly less pronounced body weight reduction (Figure 4C) and only a minor loss of body temperature during infection in comparison to controls that received siLUC (Figure 4D). Overall diminution of virus-mediated pathology under siCSF-1 influence was further corroborated by a significant reduction of cardiac troponin T serum levels as a heart-specific sign of tissue damage (Figure 4E). Cardiac troponin T at an early state of myocarditis might reflect both direct virus-induced cytotoxicity and tissue destruction by innate mediators of the immune response. In line with this, analysis of heart tissue obtained from siCSF-1 treated animals sacrificed 8 days p.i. revealed distinct differences. Histological staining of heart tissue (Figure 4F) demonstrated a profound myocarditis in siLUC-treated A.BY/SnJ mice and in contrast to that only moderate signs of myocarditis after siCSF-1 treatment. Since viral injury of cardiomyocytes provokes an inflammatory response that significantly contributes to tissue damage and functional impairment of the heart (32), next we quantified infiltration with CD45^+^ immune cells into hearts from siCSF-1 and siLUC-treated mice by flow cytometry. We found a significant reduction of infiltrating leucocytes in siCSF-1 treated mice (Figure 4G), thus indicating reduced inflammatory organ damage under suppression of the CSF-1 axis. Following up on observed systemic and heart-tissue specific responses to siCSF-1 treatment, siCSF-1 effects on cardiac performance were assessed by echocardiography during the inflammatory peak of viral myocarditis. In siLUC-treated, infected A.BY/SnJ mice, both the stroke volume and cardiac output were significantly reduced in comparison to baseline measurements (Figure 4H) and Table S1. Consistent with its heart-directed effects, siCSF-1 treatment mitigated these detrimental changes and CVB3 infection in this group resulted only in minor, non-significant reduction of cardiac performance (Table S1).

### siRNA-mediated knockdown of CSF-1 diminishes immune cell infiltration during acute viral myocarditis

**Figure 5:**
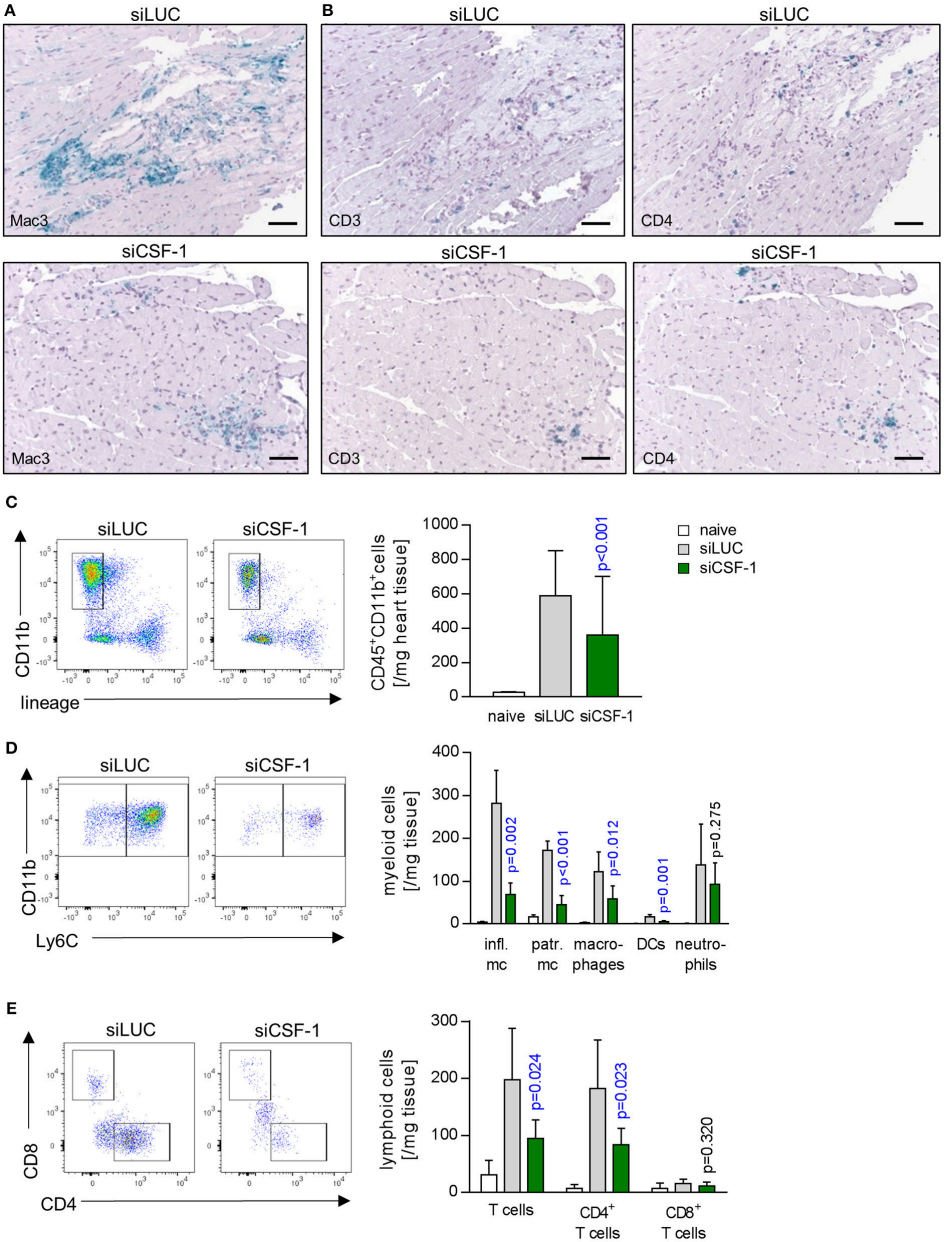
Influence of CSF-1 on immune cell infiltration into heart tissue during AVM. To further differentiate immune cell infiltration, immunohistochemical stain for **(A)** mononuclear phagocytes using antibodies directed against Mac-3 and **(B)** T-cells using antibodies directed against CD3 and CD4 were performed (scale bars = 60 μm). Further differentiation by flow cytometry (Figure S2) was performed. **(C)** Total infiltrating myeloid cells (identified as CD45^+^, CD11b^+^, lymphoid lineage^−^ life single cells) were quantified. **(D)** Myeloid cells were further differentiated according to the gating strategy depicted in Figure S2A. **(E)** Equally, lymphoid cells were further analyzed. Representative flow cytometry dot blots of siLUC- and siCSF-1-treated groups are depicted. Unpaired *t*-tests were performed between siLUC- and siCSF-1-treated groups and *p*-values are shown. Significant differences (*p* < 0.05) are marked with blue color.

As a next step, we aimed to determine whether siCSF-1 specifically influenced infiltration with monocytes/macrophages or whether other immune cells were affected as well. Immunohistochemistry using antibodies directed against marker proteins for myeloid (Mac3) and T cells (CD3 and CD4) indicated reduced infiltration of these respective immune cell populations in siCSF-1-treated mice during AVM (Figures 5A,B). These findings were corroborated by the results obtained from a quantitative flow cytometry-based analysis of the different immune cell populations in infected mouse hearts. We detected 756 ± 63 CD11b^+^/lineage^−^ cells in siLUC- vs. 273.3 ± 30.8 CD11b^+^/lineage^−^ cells/mg heart tissue in siCSF-1 treated mice (*p* < 0.0001) and 198 ± 34 T cells in siLUC- vs. 95 ± 12 T cells/mg heart tissue in siCSF-1 treated mice (*p* = 0.02; Figures 5C,E). The vast majority of infiltrating myeloid cells belonged to the pool of inflammatory monocytes (Figure 5D). Consistent with the influence of CSF-1 on monocyte recruitment and differentiation, inflammatory monocytes were highly significantly reduced in infected mouse hearts upon siCSF-1 treatment. siCSF-1 also led to a significant reduction of patrolling monocytes, macrophages and dendritic cells (Figure 5D), which might all originate from inflammatory monocytes. In correspondence to previous reports (15), the pool of invading T cells during AVM was majorly comprised of CD4^+^ T cells. Comparable to siCSF-1-induced effects on myeloid cell infiltration, we also found a significant reduction of the CD4^+^ T cell count in heart tissue upon siCSF-1-treatment (Figure 5E).

### siRNA-mediated knockdown of CSF-1 mitigates inflammatory heart tissue damage in a mouse model of experimental autoimmune myocarditis

**Figure 6:**
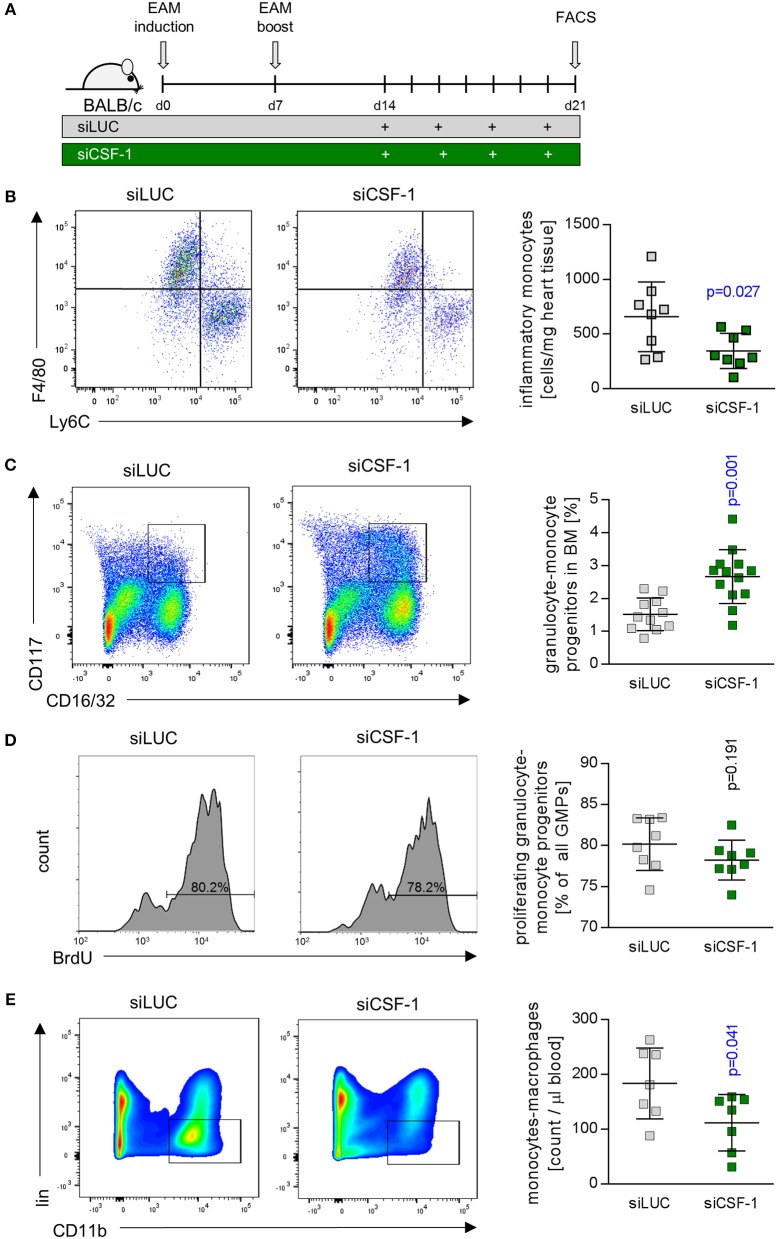
CSF-1 silencing during EAM diminishes infiltration of inflammatory monocytes into injured mouse hearts. **(A)** EAM was induced by inoculation of myosin peptide in conjunction with Freud's adjuvant and mice were boosted after 7 days. Nanoparticle-encapsulated CSF-1 siRNA #7 (Figure 3) was investigated regarding to its potential to manipulate EAM in comparison to siLUC (control). Therefore, mice were treated with 0.5 mg/kg siRNA intravenously every other day starting 14 days after EAM induction, and mice were sacrificed 21 days after the first immunization. **(B)** Representative dot plots (left) and enumeration (right) of inflammatory Ly6C^hi^ monocytes in heart tissue of siLUC- and siCSF-1-treated mice (*n* = 8). **(C)** Representative dot plots (left) and quantification of granulocyte-monocyte progenitors (GMPs) (right) found in bone marrow of siLUC- and siCSF-1-treated mice during EAM (*n* = 12). **(D)** Representative FACS plots (left) and quantification (right) of BrdU incorporation in GMPs of the bone marrow in siLUC- and siCSF-1 treated mice (*n* = 8). **(E)** Representative FACS density plots (left) and quantification of monocytes/macrophages (right) in the blood of siLUC- and siCSF-1- treated mice (*n* = 7). Unpaired *t-*tests were used. *p*-values are indicated on the graph and significant differences (*p* < 0.05) are marked with blue color.

siCSF-1 treatment impressively improved cardiac function upon mitigating the inflammatory damage response during acute viral myocarditis. A high inflammatory disease burden at the acute state might directly translate into the manifestation of a chronic functional impairment. Long-term sequela of acute myocarditis involve cardiac remodeling processes with substantial fibrosis formation and a reduction of systolic cardiac function (4). Experimental autoimmune myocarditis (EAM) represents an excellent model that enables researchers to follow inflammatory disease progression from acute to chronic states of myocardial dysfunction (40). Therefore, we investigated the pathophysiological role of CSF-1 also in EAM, and started siCSF-1 treatment upon manifestation of myosin-heavy chain-directed autoimmunity 14 days after the first immunization (Figure 6). This application strategy might also be considered as a therapeutic regime starting upon manifestation of acute disease. Similar to the AVM model, intravenous siCSF-1 nanoparticle application was repeated every 48 h for a 1-week course. First, we performed a quantitative flow cytometry-based analysis of inflammatory monocytes in the injured hearts directly after this siCSF-1 treatment period. As expected from our results obtained in AVM, we indeed found a reduced number of inflammatory Ly6C^high^ monocytes in siCSF-1-treated animals compared to the siLUC-treated controls during EAM (Figure 6B). To investigate whether CSF-1 promotes monocyte development from bone-marrow sources during EAM, we next evaluated the influence of CSF-1 knockdown on granulocyte-monocyte progenitor (GMP) cell numbers. siRNA-mediated knockdown of CSF-1 led to markedly increased numbers of GMPs in the bone marrow during EAM (Figure 6C). However, proliferation rates of GMPs did not differ significantly between siLUC- and siCSF-1-treated groups (Figure 6D). To further validate that CSF-1 knockdown leads to an arrest of progenitor cells in the bone marrow, we measured myeloid cell numbers in the blood and found reduced numbers 21 days after EAM induction (Figure 6E).

**Figure 7:**
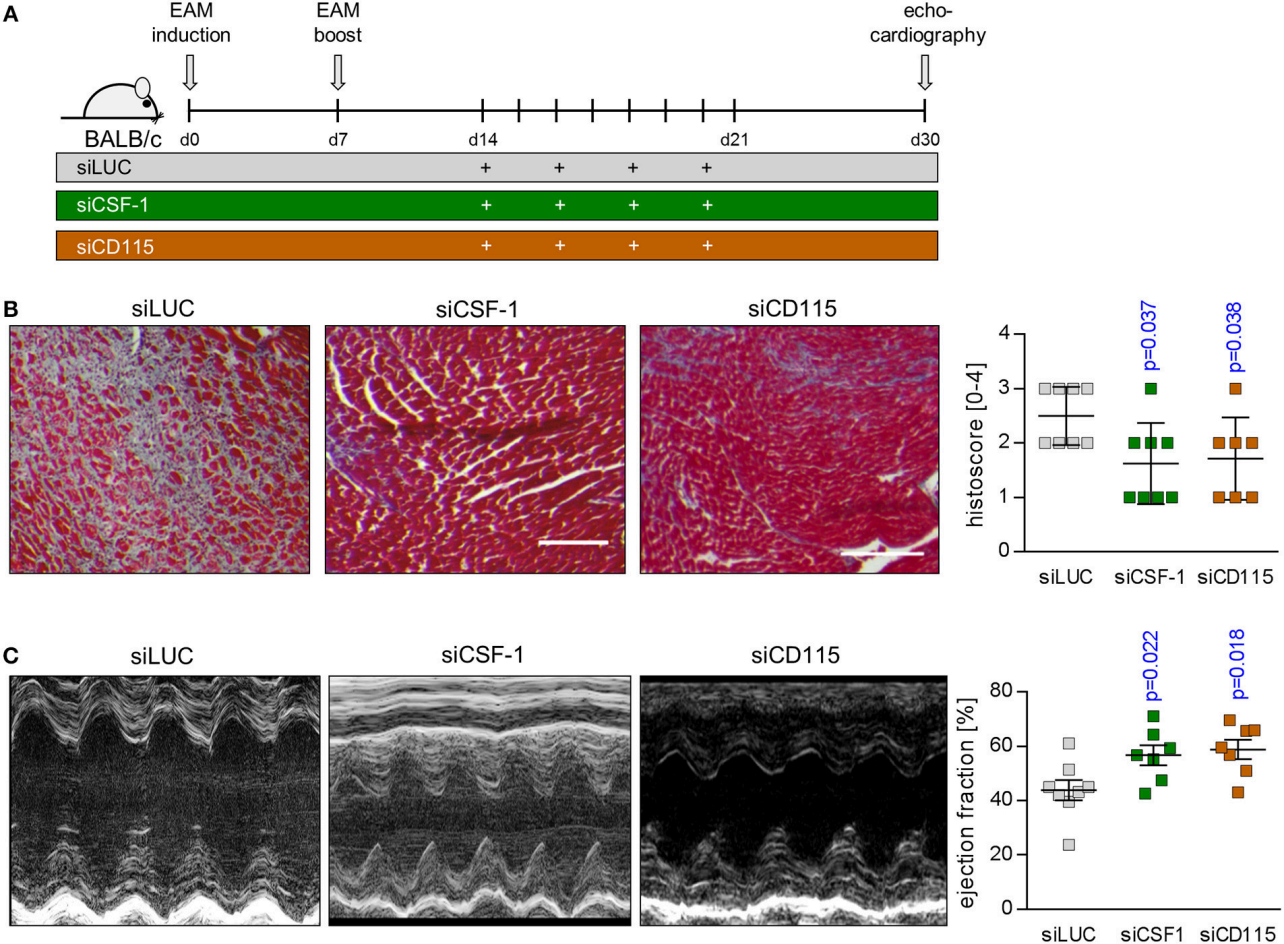
siRNA-mediated knockdown of CSF-1 during acute EAM attenuates the development of chronic disease states**. (A)** EAM induction and siRNA treatment was conducted as shown in **A**. Mice were sacrificed after 30 days. **(B)** Representative Masson's trichrome stains of heart tissue sections obtained 30 days after EAM induction are depicted (left: scale bar = 150 nm). Fibrosis was scored microscopically (*n* = 8 for siLUC and siCSF-1 as well as *n* = 7 for siCSF-1R). **(C)** Heart function was evaluated 30 days after the initial immunization by echocardiography. Representative M-mode echocardiographic images are shown during late state EAM. Calculated left ventricular ejection fraction (EF) (*n* = 8 for siLUC and siCSF-1 as well as *n* = 7 for siCSF-1) is shown. One-way-ANOVA was performed. Since ANOVA was significant, a Sidak-Holm-multiple comparison was performed. *p*-values of multiple comparison are indicated. Blue color indicates *p* < 0.05.

**Figure 8:**
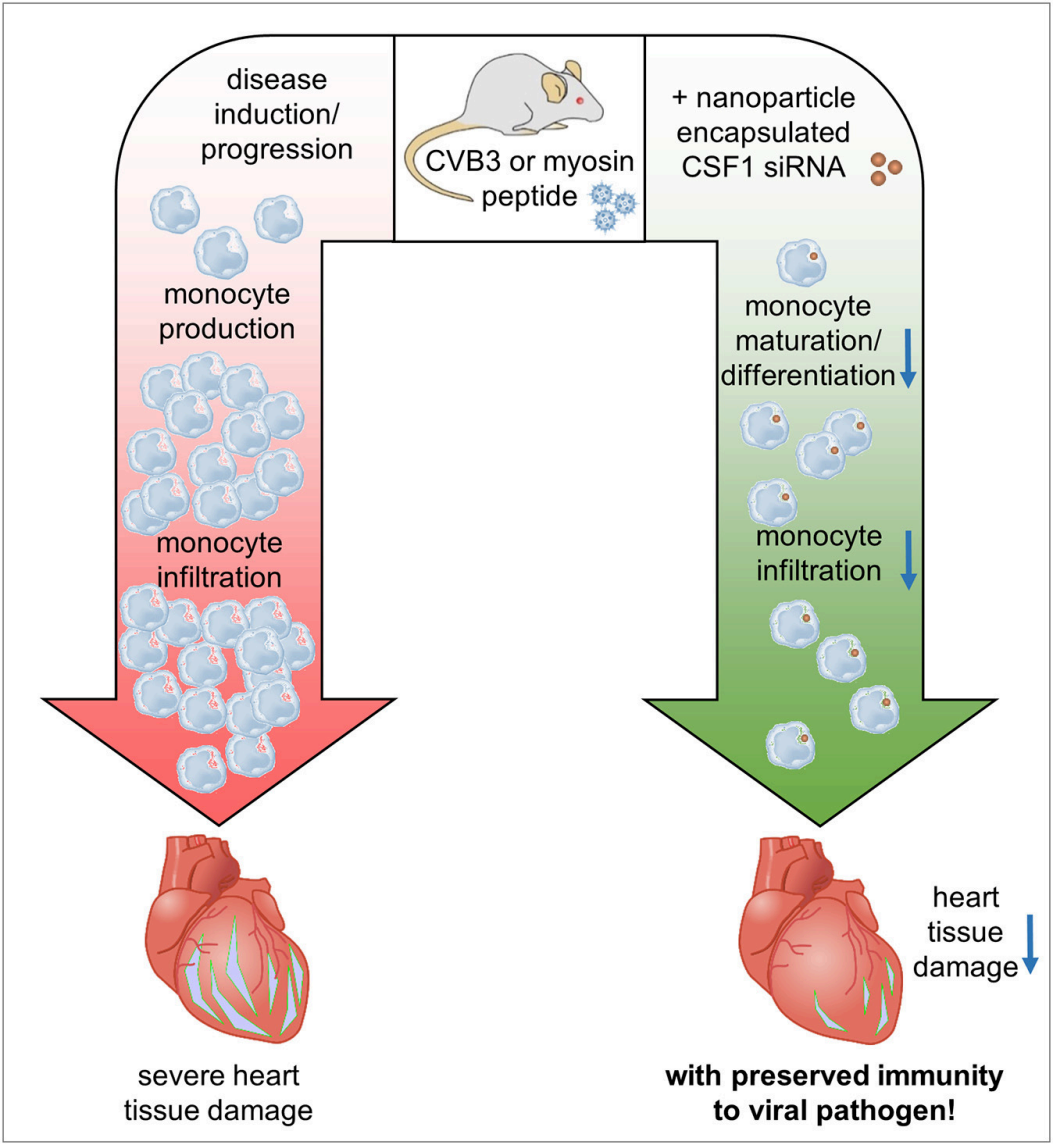
Graphical synopsis: The CSF-1 axis is induced in patients with acute myocarditis. Based on the preponderant role of CSF-1 for monocyte differentiation/maturation and the disease-modifying function of monocytes during the course of inflammatory heart disease, we investigated how silencing of CSF-1 in monocytes/macrophages using nanoparticle encapsulated siRNA influences heart tissue damage during the onset of acute viral myocarditis and upon manifestation of acute inflammation in an autoimmune myocarditis model. Silencing of the myeloid CSF-1 axis was beneficial for preventing inflammatory tissue damage in the heart and preserving cardiac function at acute and chronic disease states without compromising innate immunity's critical defense mechanisms.

Based on our finding of reduced infiltration with inflammatory monocytes upon siCSF-1 treatment, next we followed mice for a total of 30 days after EAM induction and determined the formation of fibrotic scars as an integral hallmark of cardiac remodeling at this chronic disease state (Figure 7A). Knockdown of CSF-1 production resulted in a significant reduction of fibrosis formation in the heart muscle as indicated by quantitative assessment of Masson's trichrome stains (Figure 7B). Consistent with this reduction of long-term, inflammation-mediated tissue damage in the siCSF-1 group, functional investigation of cardiac performance by echocardiography revealed an improved ejection fraction in siCSF-1-treated animals compared to siLUC-treated control animals (Figure 7C). Chronic disease in siLUC-treated mice was mirrored by a substantial reduction of cardiac contractility. As a proof of principle, we also tested whether knockdown of CSF-1R expression could exert similar effects during disease course. Therefore, upon establishment of autoimmune myocarditis mice were treated with a nanoparticle-encapsulated siRNA that specifically targets CSF-1R (33). Formation of cardiac fibrosis assessed 30 days after EAM induction was found to be diminished in comparison to siLUC-treated controls, and was reduced to similar levels as achieved by siCSF-1-treatment (Figure 7B). Likewise, echocardiographic imaging of siCSF-1R-treated animals demonstrated significant improvement of cardiac performance as indicated by higher left ventricular ejection fraction in comparison to siLUC nanoparticle-treated animals (Figure 7C). Altogether, our data demonstrate that silencing the CSF-1 axis hampers monocyte development and substantially mitigates inflammatory heart tissue damage both in a mouse model of viral and autoimmune-mediated myocarditis. Moreover, initiation of CSF-1 knockdown upon manifestation of inflammatory tissue damage attenuates the manifestation of debilitating long-term sequela of acute inflammatory injury, and this parallels in preserved systolic contractility of the heart muscle (Figure 8).

## Discussion

One of the major causes of heart failure particularly in young patients is myocarditis. Direct viral cytotoxicity stimulates infiltration and immune response activation leading to pathogen clearance and resolution of organ damage. Nevertheless, in immune-genetically predisposed individuals there is also an adverse scenario where pathogen-induced immune response activation subsequently induces overwhelming inflammatory cytokine response and detrimental immunopathology or autoimmune processes, both leading to cardiac remodeling and fibrotic scarring. It appears to be a slim line between the induction of inflammation to fight the virus and exaggerated immune responses that begin to be deleterious. We here downregulated an important branch of the innate immunity—the development of monocytes/macrophages—by siCSF-1 treatment for approximately a week during the acute phase of a viral infection, yet found no impairment on pathogen control. In fact, silencing CSF-1 production impressively mitigated acute inflammatory heart tissue damage and attenuated the development of a debilitating long-term sequela of acute inflammatory injury to the heart. This nanoparticle-mediated immune-modulation improved the course of disease both in acute viral myocarditis and autoimmune inflammatory heart disease and importantly, did not adversely influence viral burden and clearance of infectious particles. As it might be hypothesized that attenuating inflammatory activity could allow for enhanced virus-induced cell death, this is not what we observed with siCSF-1 treatment. Our finding is in line with previous data: out of 46 studies that intervened the immune response, more than 90% found no adverse effect on viral load (4). In line with this, we could recently demonstrate that inhibition of cellular proteolysis in immune cells by the immunoproteasome inhibitor ONX 0914 is highly efficient to reverse high grade AVM and this protective effect was attributed to maintained immune cell homeostasis, but not to direct antiviral aspects (14).

A.BY/SnJ mice that were used in this study for AVM are characterized by a high hereditary susceptibility to CVB3-induced cardiomyopathy (6, 15, 41). Upon viral infection of cardiomyocytes, heart tissue injury during the first days is mainly attributed to direct virus-induced cytotoxicity, while the activation of local type I interferon responses (T1IFN) is substantially hampered in this strain (7, 8). This phase of ongoing viral replication is accompanied and succeeded by the recruitment of cells of the innate immune response such as NK cells and monocytes (42). Later on, infiltration with immune cells of the adaptive immune response such as T and B cells follows (15). Analysis of the composition of leukocytes in the heart revealed that 8 days after viral infection a general reduction in invading immune cells was observed which suggests that hampering early responders, such as monocytes e.g., by reduced production of lymphocyte-attracting chemokines might dampen the infiltration and activation of subsequent populations as well. Although we found an overall reduction of CD4^+^ T cells in the heart in siCSF-1 treated mice, we cannot conclude on the influence of silencing the CSF-1 axis on CD4^+^ T cell differentiation e.g., into regulatory T cells or Th1 and Th17 cells. From our data we cannot conclude on possible additional effects e.g., of regulatory CD4^+^ T cells that are known to mitigate the inflammatory tissue damage in the heart (43, 44). CSF-1 facilitates myeloid cell differentiation, monocyte survival, and macrophage proliferation. It was recently shown that CSF-1R also plays a role in splenic monocytopoiesis (33). Monocytes are also important producers of CSF-1 themselves. Hume and MacDonald suggested that modulation of the CSF-1 axis may be beneficial under pathological conditions (45). We here show that siRNA-mediated knockdown of CSF-1 in monocytes and its imminent precursors leads to a significant reduction of inflammatory Ly6C^hi^ monocyte numbers in the inflamed heart. We speculate that this observation results from arresting the CSF-1-dependent monocyte/myeloid cell development at precursor stages. This hypothesis is supported by detection of increased GMP cell numbers in the bone marrow in siCSF-1-treated animals during EAM. Although increased cell numbers may also arise from increased proliferation rates, this situation seems to be unlikely, since the percentages of proliferating GMPs in the bone marrow of siCSF-1- and siLUC-treated animals did not differ. Consistently, the numbers of myeloid cells were reduced in the blood stream of siCSF-1-treated animals during EAM. In agreement with this and the fact that other than in siLUC-treated mice enhanced monocyte counts were not observed in the blood in siCSF-1-treated mice during AVM (data not shown), we found a reduction of CSF-1R abundance in the spleen during AVM in the siCSF-1 group. The spleen itself is a source for monocytes with local production and from which they are recruited to the site of inflammation (34). CSF-1R is expressed throughout the mononuclear phagocyte system, which is primarily composed of monocytes and macrophages (22, 46). A reduction of CSF-1R expression might also be indicative for reduced numbers of monocytes and macrophages. These findings further underscore our assumption of a halted monocyte/macrophage production due to CSF-1 knockdown during inflammation. Mice that carry a deleterious mutation in CSF-1 develop characteristic skeletal malformation, caused by defective osteoclasts. In the context of an inflammatory disease, such as atherosclerosis however, deletion of CSF-1 results in dramatically reduced atherosclerotic plaque size (47, 48). After myocardial infarction an upregulation of CSF-1 is observed in ischemic areas for more than 5 days after the injury (49). In this context, CSF-1 also appears to exhibit indirect effects by regulating chemokine production (49). In the absence of CSF-1, GM-CSF controls the differentiation of selected macrophage subsets and may lead to an enhancement of macrophage lineage numbers (50). Under certain conditions, GM-CSF induces an inflammatory program marked by increased IL-1, IL-6 and TNF-α secretion, whereas the presence of CSF-1 suppresses the production of pro-inflammatory signals (51). Others have reported that administration of CSF-1 in EAM between days 21 and 29 after disease induction ameliorated cardiac fibrosis and left ventricular dysfunction by preventing the accumulation of fibroblasts (52). Notably, no effects were observed in the above mentioned study when CSF-1 was administered at later stages. These conflicting results stress the importance of timing in CSF-1 modulation. The reduced numbers of inflammatory monocytes due to silencing myeloid CSF-1 from days 14 to 21 observed in this study may outweigh the beneficial effects of CSF-1 in the inflamed heart at these early stages. In addition, macrophages have been shown to release pro-fibrotic cytokines such as TGF-β, which causes the differentiation of fibroblasts to myofibroblasts and a massive deposition of extracellular matrix proteins such as collagens (53). Since we see a reduction of myeloid cells during myocarditis in response to siCSF-1 treatment and this includes macrophages, we propose that thereby achieved reduction of pro-fibrotic signals most likely contributes to lower scar formation as observed at advanced states of EAM.

It has been reported that other cell types beyond cells of the myeloid lineage are capable of CSF-1 expression under inflammatory conditions, including fibroblasts and endothelial cells (54, 55). These cells may also influence monocyte production/proliferation during myocarditis. Nevertheless, at the acute state of virus-mediated myocarditis, we observed that Mac-3 positive cells like monocytes/macrophages, which infiltrate the heart, may represent the main producers of CSF-1. Many studies using different pathogenic models of bacterial, viral and fungal infections have highlighted the importance and requirement of TNF- and NO-producing monocytes as facilitators of the resolution of an infection (56). Also, during inflammation bone-marrow derived monocytes can mature into macrophages (56) and macrophage-depletion results in increased virus titers in infected mouse hearts (17). Therefore, it was somewhat surprising at the first glance that depletion of monocytes upon siCSF-1 treatment actually improved virus-mediated pathology. We found no experimental evidence that virus dissemination, control and elimination had been adversely affected by such immune-modulating intervention. Since virus titers were not significantly influenced by siCSF-1-treatment, the impressively reduced inflammatory injury and preserved cardiac function in this group is not influenced by direct virus-mediated effects. These data suggest that virus-induced inflammation can be segregated from pathways that promote and limit virus infection and CSF-1-induced monocyte maturation. Monocytes can be directly activated by CVB3 infection resulting in the production of pro-inflammatory cytokines (57). Thereby, these cells can contribute to a strong inflammatory response and eventual tissue damage in the myocardium. Further experimental evidence for an adverse function of myeloid immune cells comes from macrophage depletion studies, where myocardial injury and formation of cardiac fibrosis were substantially diminished despite and in clear contrast to increased viral burden during AVM (17). Interestingly and in line with our findings in siCSF-1 treated A.BY/SnJ mice, macrophage depletion did not adversely affect clearance of infectious virus particles (17). We conclude that attenuated innate immune cell mobilization and hampered CSF-1-driven differentiation of innate myeloid cells in siCSF-1-treated mice directly suppresses cytotoxicity induced by cytokine production and/or infiltration with lymphocytes in viral myocarditis. Although there is a clear causal relationship between T1IFN-mediated suppression of viral load in infected cardiac cells and attenuation of inflammation and chronic tissue damage (8, 32), several publications including work of our group support the concept that—independent of direct virus-induced cell injury—particularly monocytes and macrophages are important players in inflammation and chronic organ damage in response to Coxsackievirus infection (4, 14, 58, 59).

Taken together, modulation of the CSF-1 axis in the myeloid cell lineage with siRNAs at early stages has beneficial acute and long-term effects in both viral and autoimmune myocarditis. Our data support the notion that particularly infiltrating myeloid cells contribute to acute and chronic functional impairment in inflammatory heart disease. Since pathogen control was not influenced upon suppression of the CSF-1 axis in myeloid cells, this study yields important insights for translating the pathophysiological role of CSF-1 from animal models to putative novel therapeutic targets for patients with inflammatory heart disease.

## Ethics statement

The requested information is provided in the material and method section of the manuscript.

## Author contributions

AB and FL conceptualization. AB, FL, IM, CG, MK, MS, BM, JH, and KK methodology. IM, CG, KK, MS, AH, VE, H-PV, DA, JH, and MK investigation. IM and CG formal analysis. CG and IM visualization. FL, AB, IM, and CG writing-original draft. HK drafting/revising critically. AB, FL, and HK funding acquisition. AB and FL supervision.

### Conflict of interest statement

The authors declare that the research was conducted in the absence of any commercial or financial relationships that could be construed as a potential conflict of interest.
