# Supplementary figures and images for: Silencing the CSF-1 Axis Using Nanoparticle Encapsulated siRNA Mitigates Viral and Autoimmune Myocarditis
Source: Front Immunol. 2018 Oct 8;9:2303. doi: 10.3389/fimmu.2018.02303 (PMC6186826; doi:10.3389/fimmu.2018.02303)

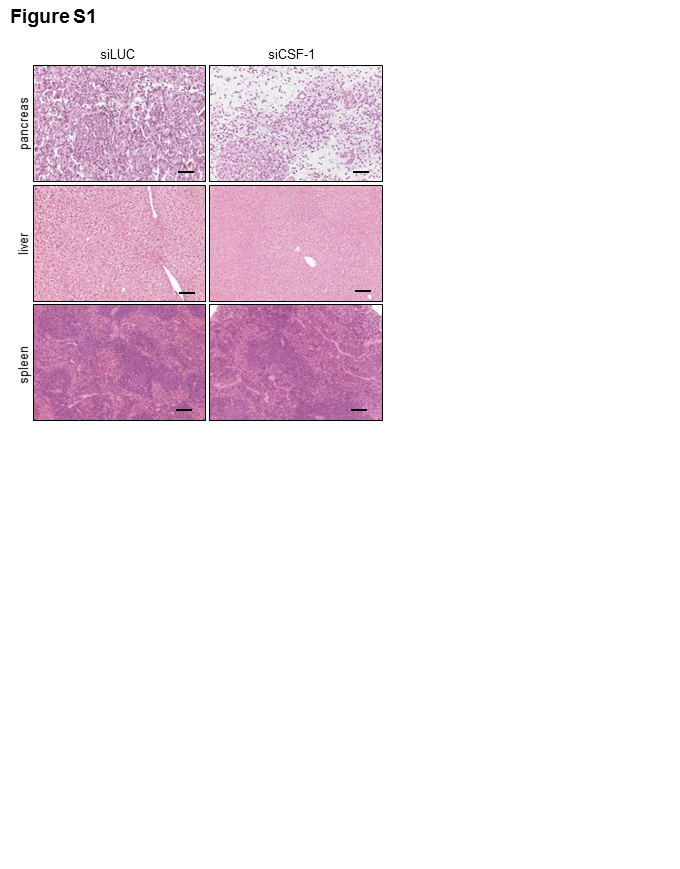

Supplement: Supplementary file 1 [file Image_1.TIF]

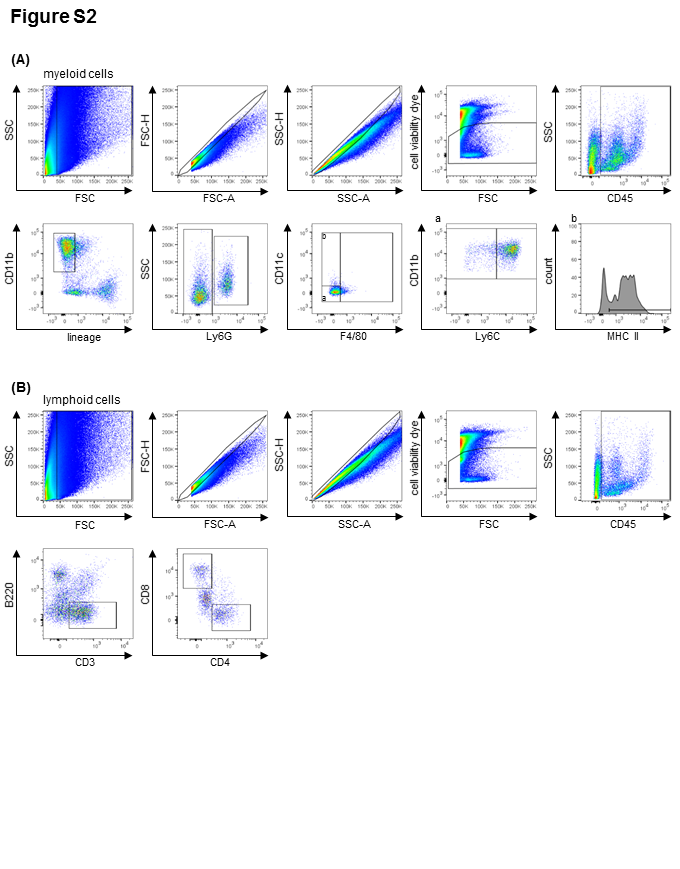

Supplement: Supplementary file 2 [file Image_2.TIF]
