# Supplementary material for: Silencing the CSF-1 Axis Using Nanoparticle Encapsulated siRNA Mitigates Viral and Autoimmune Myocarditis
Source: Front Immunol. 2018 Oct 8;9:2303. doi: 10.3389/fimmu.2018.02303 (PMC6186826; doi:10.3389/fimmu.2018.02303)
Supplement: Supplementary file 3 [file Data_Sheet_1.PDF]

Silencing the CSF-1 axis using nanoparticle encapsulated siRNA mitigates viral and autoimmune myocarditis.

Ingmar Sören Meyer [1,2]*, Carl Christoph Goetzke [3,4]*, Meike Kespohl [3,4], Martina Sauter [5], Arnd Heuser [6], Volker Eckstein [7], Hans-Peter Vornlocher [8], Daniel G. Anderson [9,10,11], Jan Haas[1,2], Benjamin Meder[1,2], Hugo Albert Katus [1,2], Karin Klingel [5], Antje Beling [3,4*,§§] and Florian Leuschner [1,2,*,§§]

Supplemental Information

Figure S1

CSF-1-directed siRNA treatment has no deleterious impact on organ homeostasis of pancreas, liver and spleen. The innate immune system is essential for counteracting CVB3 infection in mice. To preclude any putative disease-deteriorating impact of CSF-1 siRNA treatment on viral infection in other organs, tissue sections were obtained from pancreas, liver and spleen 8 days after virus inoculation. Representative micrographs are shown from a total of n=7 siLUC and n=8 siCSF-1-treated mice. Scale bars: pancreas = 60 µm; liver and spleen = 120 µm. We found no signs of altered tissue damage or infiltration with immune cells in these organs under siCSF-1 influence.

Figure S2

Gating and identification of different myeloid and lymphoid cells in heart tissue-derived single cell suspensions during AVM. (A) Monocytes were identified as Fixable Viability Dye^{low}, CD45.2^{+}, CD11b^{high}, Lin^{-} (B220, CD90.2, CD49, NK-T/NK Cell Antigen, Ter-119)$^{-}$, Ly6G^{low}, F4/80^{-} and CD11c^{-} and further differentiated according to Ly6C-expression. Inflammatory monocytes express high levels of Ly6C and patrolling/stationary monocytes express low levels of Ly6C. Macrophages were identified as Fixable Viability Dye^{low}, CD45.2^{+}, CD11b^{high}, Lin^{-}, Ly6G^{low}, , F4/80^{+} and CD11c$^{-/+}$. Dendritic cells were identified as Fixable Viability Dye^{low}, CD45.2^{+}, CD11b^{high}, Lin^{-}, Ly6G^{-}, F4/80^{-}, CD11c^{+} and MHC II^{+} (compared to isotype control). Neutrophils were identified as Fixable Viability Dye^{low}, CD45.2^{+}, CD11b^{high}, Lin^{--}, Ly6G^{high} and SSC^{high}. (B) T-cells were gated as Fixable Viability Dye^{low}, CD45.2^{+}, B220^{-} , CD3^{+} and either CD4^{+} or CD8^{+}.

Table S1

	siLUC		siCSF-1	
	baseline	AVM	baseline	AVM
heart rate [bpm]	424 ± 38	423 ± 48	405 ± 45	412 ± 41
LV-d [mm]	3.2 ± 0.4	2.9 ± 0.4*	3.2 ± 0.3	3.0 ± 0.4
LV-s [mm]	2.0 ± 0.3	1.9 ± 0.3	2.1 ± 0.4	2.0 ± 0.4
Vol-d [µl]	30.0 ± 6.1	21.0 ±4.1*	29.2 ± 5.5	23.6 ± 5.2*
Vol-s [µl]	9.9 ± 2.7	7.0 ± 2.2*	11.3 ± 4.0	9.1 ± 3.4
trace EF [%]	67.2 ± 5.1	67.1 ± 8.9	61.6 ± 10.0	62.2 ± 9.2
SV [µl]	20.1 ± 4.0	14.1 ± 3.0*	17.9 ± 4.0	14.5 ± 3.1*
CO [ml/min]	8.5 ± 1.9	6.0 ± 1.6*	7.7 ± 2.4	6.0 ± 1.6

Analysis of cardiac function upon CSF-1-directed siRNA treatment during AVM. Cardiac function was assessed by echocardiography prior to CVB3 infection in A.BY/SnJ mice (baseline) by an experienced and blinded investigator. Mice were allocated to respective groups: siLUC – siRNA directed against luciferase; siCSF-1 – siRNA directed against CSF-1. In all CVB3-infected mice, echocardiography was repeated 8 days after CVB3 infection; siLUC n = 16; siCSF-1 n = 17 mice). Data were analyzed regarding putative alteration during AVM in the respective treatment groups (day 8 after infection vs. baseline measurements of the same cohort). Data are presented as mean values ± SD. * indicates significant differences ($p < 0.05$) compared to baseline measurements (paired student‘s *t* test). bpm: beats per minute; LV-d: left ventricle internal diameter at diastole; LV-s: left ventricle internal diameter at systole; Vol-d: end-diastolic volume; Vol-s: end-systolic volume; trace EF: trace ejection fraction; SV: stroke volume; CO: cardiac output.
